# HCV Replicon Systems: Workhorses of Drug Discovery and Resistance

**DOI:** 10.3389/fcimb.2020.00325

**Published:** 2020-06-30

**Authors:** Shaheen Khan, Shalini Soni, Naga Suresh Veerapu

**Affiliations:** Virology Section, Department of Life Sciences, Shiv Nadar University, Gautam Buddha Nagar, India

**Keywords:** hepatitis C virus, replicon, direct-acting antiviral, resistance-associated substitution, drug discovery, drug resistance, genotype

## Abstract

The development of direct-acting antivirals (DAAs) has revolutionized the state-of-the art treatment of HCV infections, with sustained virologic response rates above 90%. However, viral variants harboring substitutions referred to as resistance-associated substitutions (RASs) may be present in baseline levels and confer resistance to DAAs, thereby posing a major challenge for HCV treatment. HCV replicons have been the primary tools for discovering and evaluating the inhibitory activity of DAAs against viral replication. Interest in replicon systems has further grown as they have become indispensable for discovering genotype-specific and cross-genotype RASs. Here, we review functional replicon systems for HCV, how these replicon systems have contributed to the development of DAAs, and the characteristics and distribution of RASs for DAAs.

## Introduction

The molecular cloning of hepatitis C virus (HCV) in 1989 led to advances in fundamental research to fully decipher the virus life cycle and develop treatments for its eradication (Hoofnagle et al., 1986; Choo et al., 1989; Scheel and Rice, 2013; Alazard-Dany et al., 2019). HCV is a member of the *Flaviviridae* family that also includes the causative viruses of West Nile, Dengue, Yellow fever, and Zika diseases. Following acute HCV infection, 70% of individuals develop chronic hepatitis C. The Word Health Organization (WHO) estimates that ~71 million people have chronic hepatitis C globally (World Health Organization, 2017). Chronic hepatitis C can lead to cirrhosis and hepatocellular carcinoma (HCC), followed by death in about 5% of individuals (Stanaway et al., 2016). Currently available HCV treatments have successful elimination rates above 95% (Holmes et al., 2019). Successful treatment is confirmed by the absence of HCV RNA via polymerase chain reaction (PCR) assays, with an assessment at 12 weeks after the end of treatment, thereby indicating sustained virologic responses (SVR). There are currently no prophylactic or therapeutic vaccines against hepatitis C (Roingeard and Beaumont, 2020).

Improvement in the treatment and SVR against HCV was facilitated by the discovery of pangenotypic direct-acting antivirals (DAAs), with replicon systems playing significant direct roles. HCV replicons are subgenomic RNA molecules that are capable of autonomously replicating in hepatoma cells. The replicons are primary composed of NS3-to-NS5B sequences that encode enzymes essential for viral replication (Lohmann et al., 1999; Bartenschlager, 2002, 2006; Lohmann, 2009). The underlying mechanism relies on DAAs targeting viral enzymes that are not expressed by hepatoma cell genomes, and they should also be effective in treating HCV-infected patients, in addition to likely inducing minimal side effects. However, the high genetic variation of HCV poses a major challenge for developing pangenotypic DAAs. The high viral diversity is partly due to high error rates from HCV replication (Ogata et al., 1991; Neumann, 1998; Geller et al., 2016). Resistance to DAAs arises from mutations in NS3-to-NS5B sequences that encode viral enzyme targets of DAAs. Consequently, selection and persistent replication of variants harboring amino acid substitutions that confer resistance to DAAs are major causes for reduced treatment efficacy (Wyles and Luetkemeyer, 2017). Hence, preclinical assessments of drug resistance profiles have gained important roles in the development of DAAs. Further, replicon systems have been used to study treatment-related resistance associated substitutions (RASs) that confer resistance to DAAs across genotypes (GTs) and subtypes (Ng et al., 2017a; Han et al., 2019).

## Virology

**Figure 1:**
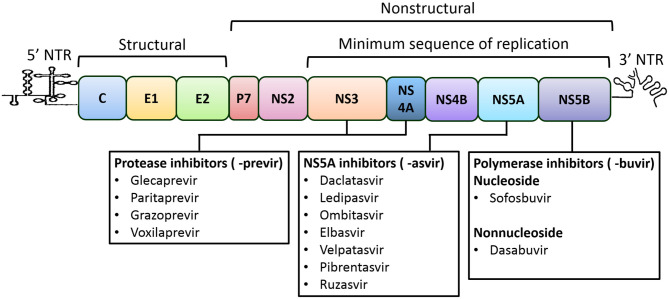
HCV genome organization and direct-acting antivirals for the treatment of HCV Infection. The HCV genome is consisted of single open reading frame (ORF) that is flanked by 5' and 3' non-translated regions (NTRs). The IRES present at the 5' NTR mediates the translation of the ORF leading to the formation of a polyprotein, which is further processed into three structural (core, E1 and E2) and seven nonstructural proteins (P7, NS2, NS3, NS4A, NS4B, NS5A, and NS5B). The NS3-to-NS5B coding region is the minimal sequence required for RNA replication (Lohmann et al., 1999). Recommended direct-acting antivirals are listed below and include the NS3/4A (or protease; names end in -previr) inhibitors, the NS5A inhibitors (names end in -asvir), and the NS5B (or polymerase) inhibitors of nucleoside and non-nucleoside (names end in -buvir).

HCV is an enveloped virus comprising an ~9.6-Kbp-long single-strand RNA genome with positive polarity. The genome constitutes a 5' non-translated region (NTR), and a single large open reading frame, followed by a 3' NTR. An internal ribosomal entry site (IRES) present at the 5' NTR translates the ORF to a large polyprotein of about 3,000 amino acids length that is then processed by viral and cellular proteases into three structural and seven non-structural proteins (Figure 1) (Moradpour et al., 2007; Alazard-Dany et al., 2019). The structural protein core forms the virus capsid. E1 and E2 are envelope transmembrane glycoproteins that aid in receptor-mediated endocytosis for viral entry (Bartosch et al., 2003). The P7 protein forms an ion channel in the endoplasmic reticulum (ER) and plays a role in viral infection. Nonstructural proteins, NS2, NS3, NS4A, NS4B, NS5A, and NS5B, act together to form replication complexes on membranous webs derived from the ER (Romero-Brey et al., 2012). NS2 is a cysteine protease that autocatalyzes the polyprotein precursor cleavage between NS2 and NS3 (Grakoui et al., 1993). NS3 also exhibits cysteine and serine protease activities that cleave NS4A-NS5B at junction regions to release individual protein components and also act as a viral helicase, while NS4A is a co-factor of NS3 (Tomei et al., 1993; Lin et al., 1994). NS4B is an integral membrane protein that aids in the formation of the viral replication complex (Egger et al., 2002). NS5A is a membrane phosphoprotein that permits viral binding and assembly of the replication complex (Tanji et al., 1995). NS5B (an RNA-dependent RNA polymerase) directs transcription of the (+) strand for production of the (-) strand that becomes the template for the new (+) strand genomes (Behrens et al., 1996; Lohmann et al., 1997). Given their essential role, the non-structural proteins of replication complex are the targets of DAAs, which can disrupt specific steps in the replication, have now become the core of the HCV treatment strategies (Lontok et al., 2015).

## HCV Genetic Heterogeneity and the Emergence of DAA Resistance

Sequencing and phylogenetic analyses of HCV isolates from different geographic regions have revealed that HCV can be classified into eight genotypes (GTs 1–8) and 86 subtypes that differ at the nucleotide (nt) level by 30 and 15%, respectively (Hedskog et al., 2019). The appearance of HCV in at least eight GTs has important implications for HCV treatment, as differences exist in achieved SVR rates against different GTs using various regimens (Mangia and Mottola, 2012). Further, the two inherent replication features of HCV can contribute to suboptimal responses to DAAs at the patient level, including their: (a) high error rate during replication and (b) high virion turnover. Due to the lack of NS5B exonuclease activity, HCV replicates with an error rate of 10^−3^ to 10^−5^ mutations per nt per round of replication and reproduce with an estimated 10^10^-10^12^ virion turnover per day in an infected individual (Ogata et al., 1991; Neumann, 1998; Geller et al., 2016). The resultant large reservoir of genetic variants circulating at a given time in an infected patient is classically referred to as a “quasispecies” (Perales et al., 2015). The majority of variants are unable to replicate due to deleterious or lethal effects of the new mutations or are otherwise cleared by the host immune system. However, some variants with beneficial mutations are continuously selected upon due to evolutionary advantages or better adaptation to adverse conditions, such as the presence of a DAA (Agarwal et al., 2018; Singh et al., 2020). Consequently, natural variants with various levels of susceptibility to DAAs may exist within patients and can be selected after DAA exposure (Ahmed and Felmlee, 2015). The selection of variants may then confer resistance to DAAs and treatment failure.

Following the discovery of HCV, numerous attempts have been made to recapitulate viral life cycles using *in vitro* cell culture systems. The first significant development was the establishment of a “subgenomic replicon” system wherein replicon cell lines were isolated that persistently harbored autonomously replicating HCV non-structural genes at appreciable levels (Lohmann et al., 1999). These replicons generally lack the viral structural genes involved in capsid formation and are hence subgenomic. The subsequent isolation of more efficient replicons paved the way for understanding HCV replication, the molecular interplay between viruses and cells, and the identification and development of inhibitors that are effective against HCV replication. The second significant breakthrough was the establishment of the infectious “HCV cell culture” system (HCVcc) based on the JFH-1 wild type isolate (GT2a) that exhibits all stages of the virus life cycle (Kato et al., 2001; Wakita et al., 2005). Further advances using JFH-1 HCVcc were made by generating a highly infectious GT2a J6/JFH1 chimera (Lindenbach and Rice, 2005). The repository of infectious HCVcc systems was expanded to include GT1a (Yi et al., 2006), GT1b (Pietschmann et al., 2009), GT2b (Ramirez et al., 2014) GT3a (Saeed et al., 2013), and 6a (Pham et al., 2018). HCVcc systems of intergenotypic are also developed (reviewed in Ramirez and Bukh, 2018). Indeed, infectious HCVcc systems have paved the way for a deeper understanding of HCV biology, including the identification of DAA-resistant variants in the context of infectious viral life cycles (Ramirez et al., 2016; Serre et al., 2016; Jensen et al., 2019).

Although infectious HCVcc systems allow the more complete study of complete viral replication cycles and understanding of genotype-specific pathogenesis, HCV replicon technology continues to be critical due to its ease of use in the discovery of candidate DAAs, and also in the identification and evaluation of RASs to improve the effectiveness of HCV treatments. Here, we provide a summary of different aspects of the HCV replicon systems and our current knowledge of RASs associated with resistance to DAAs.

## Replicon Systems

### Structure and Development of the Classical Bicistronic Replicon

**Figure 2:**
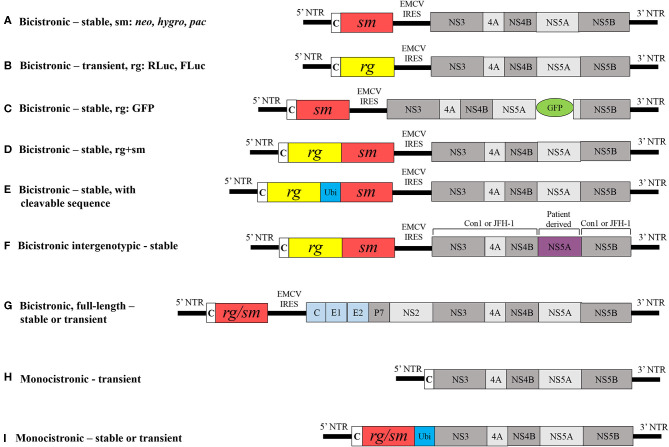
Schematic comparison of different HCV replicons. **(A)** Classical bicistronic subgenomic replicon comprised 5' NTR, first 16 codons of core, *neo* gene, a selectable marker followed by EMCV IRES, HCV NS3-to-NS5B coding region and HCV 3' NTR; Other selectable markers less widely used are *hygro* and *pac*. Modified bicistronic replicons may carry either: **(B)** a *Renilla* luciferase (RLuc) or firefly luciferase (FLuc) (reporter gene) instead of a selectable marker, **(C)** a GFP at certain positions within NS5A, **(D)** a reporter gene that is fused with a selectable marker, **(E)** ubiquitin, a host cell cleavable sequence fused in-frame between a reporter and selectable marker, **(F)** a non-structural sequence derived from patient in Con1 or JFH-1 replicons genetic background or **(G)** an entire HCV ORF, core-to-NS5B and 3' NTR. Monocistronic replicons are composed of **(H)** HCV 5' NTR and first 12 codons of core followed by NS3-to-NS5B coding sequences and 3' NTR and **(I)** ubiquitin in-frame with either a reporter gene or selection marker. NTR, non-translated region; Ubi, ubiquitin, rg, reporter gene; sm, selection marker; neo, gene encoding neomycin phosphotransferase; hygro, hygromycin phosphotransferase; PAC, puromycin N-acetyltransferase; c, core; EMCV IRES, internal ribosomal entry site from encephalomyocarditis virus; GDD, amino acid motif in the catalytic site of RNA polymerse; GFP, green fluorescent protein. Viral and non-viral coding sequences are marked by rectangles; non-coding regions are indicated by horizontal lines.

Lohmann et al. successful proof-of-principle demonstration that the minimal HCV replication and translation machinery require the NTRs and the NS3-to-NS5B coding sequences forms the basis for HCV replicon technology (Lohmann et al., 1999; Bartenschlager, 2002). Functional replicons have been established for different GTs and several subtypes have now become indispensable tools for preclinical DAA discovery (Fourati and Pawlotsky, 2015). Consensus 1 (Con1), the first HCV replicon that was generated from the consensus GT1b isolate sequence consists of two gene clusters, each with an independent cistron: (i) the HCV 5' NTR and the first 16 codons of the core gene fused in-frame with the selectable neomycin phosphotransferase gene (*neo*), and (ii) the IRES of the encephalomyocarditis virus (EMCV) that directs the translation of fused HCV NS3-to-NS5B coding sequence, and the HCV 3' NTR (Figure 2A). A replicon cloned downstream of the T7 promoter in a plasmid serves as template to generate bicistronic transcripts. Huh7 cells that are then transfected with the bicistronic transcripts are propagated for several cell division cycles in the presence of the aminoglycoside antibiotic G418. The *neo* gene that confers resistance to the cytotoxic drug G418 then enabled the selection of “stable replicon cells” that support the autonomous replication of Con1 transcripts, thereby constituting a stable HCV replicon assay. The Con1 replicon carrying the NS5B GND amino acid motif (GND in place of GDD results in loss of NS5B polymerase activity) has been used as a negative control for viral replication. Efficiency of colony formation could then be quantified as the number of selected colonies per microgram of transfected RNA. Northern blot analysis of total RNA isolated from stable Con1 replicon cells that were treated with actinomycin D (an inhibitor of DNA-directed, but not RNA-directed RNA synthesis) could then detect HCV (+)- and (−)-strand RNA (Lohmann et al., 1999; Blight et al., 2000).

The G418-resistant Con1 replicon cells that were generated persistently carried replicating HCV RNAs, albeit with low colony formation efficiency and viral RNA levels that reflected adaptive constraints in the Huh7 cell environment. Sequencing of the viral RNAs from isolated replicon cell clones then revealed key mutations localized to different non-structural genes. Inclusion of these mutations conferred the original Con1 replicon with increased colony formation efficiency, and this phenomenon subsequently was termed ‘adaptive mutation.’ Specifically, Con1-based replicons carrying two adaptive mutations located at NS3 E176G and T254I, and one at NS5A S225P (Krieger et al., 2001) or NS4B K129T (Lohmann et al., 2003) conferred synergistically enhanced RNA replication. In addition, Blight et al. (2000) identified S232I as a key adaptive mutation localized in NS5A that appears to correspond to highly efficient replication. However, the GT1b HCV-N replicon was observed to replicate efficiently in Huh7 cells, even in the absence of adaptive mutations (Ikeda et al., 2002). Moreover, two independent studies observed that NS3 P470L and NS5A S232I mutations synergistically enhance H77-based replication of the GT1a replicon (Grobler et al., 2003; Yi and Lemon, 2004). In particular, the NS5A S232I mutation played a key role in expanding the repertoire of efficient non-GT1 replicon systems (Saeed et al., 2012; Peng et al., 2013; Wose Kinge et al., 2014; Yu et al., 2014). Recently, it is shown that Sec14L2 expressing Huh7.5 cells supported replication of natural HCV isolates in cell culture (Saeed et al., 2015). The exact mechanism underlying how adaptive mutations confer enhanced replication of HCV RNAs is not completely understood. A recent study showed that S232I reduces the hyperphosphorylation of NS5A and disrupts the NS5A interaction with human vesicle associated proteins, thereby negatively regulating viral replication and contributing to the adaptive phenotype (Evans et al., 2004). Another recent study has shown that adaptive mutations could prevent a cellular lipid kinase, phosphatidylinositol 4-kinase IIIα (PI4KA) activation, which create a permissive membrane microenvironment in hepatoma cells. Further, inhibition of PI4KA activity was found to promote replication of unadapted viral isolates (Harak et al., 2017). Since the first report, HCV replicons have been further modified to be more suitable for studying viral RNA replication and to promote drug discovery. A few of the various HCV replicon derivatives are described in further detail below.

### Other Bicistronic Replicons and Modifications

The molecular structure of the classical replicon was used to establish the GT1b (Ikeda et al., 2002) GT2a (Kato et al., 2003), GT3a (Saeed et al., 2012), GT4 (Saeed et al., 2012; Peng et al., 2013), GT5a (Wose Kinge et al., 2014) and GT6a (Yu et al., 2014; Camus et al., 2018) replicon systems. In all of these systems, *neo* was used to select stable cell lines. Another selection marker that was less frequently used was puromycin N-acetyltransferase (Liang et al., 2005) and hygromycin phosphotransferase (Sir et al., 2012) (Figure 2A). Generating G418-resistant stable cell lines is time consuming, taking up to 3–4 weeks, and is dependent on the replication capacity of the replicon. To produce a rapid and direct means of analyzing transient replication capacity, *neo* was replaced by firefly luciferase (FLuc) or *Renilla* luciferase (Rluc) reporter genes in the replicon, thereby eliminating the need for selection and allowing analysis of replicon RNA replication over 72 h of post-transfection via a transient HCV replicon assay (Figure 2B) (Krieger et al., 2001; Lohmann et al., 2001; Camus et al., 2018). Other reporter gene alternatives include β-lactamase or green fluorescent protein (GFP) (Murray et al., 2003). The Con1 replicon carrying an in-frame insertion of GFP at certain positions within the NS5A gene permits the direct visualization of active replication complexes in real time (Figure 2C) (Moradpour et al., 2004). However, the insertion of GFP reduces the replication capacity of replicons by about 100-fold relative to the parental replicon without GFP (Appel et al., 2005). The most widely used replicons comprising fused RLuc and *neo* genes allows the simultaneous selection of hepatoma cells while luciferase activity provides direct evaluation of RNA replication levels (Figure 2D) (Krieger et al., 2001; Peng et al., 2013; Yu et al., 2014; Camus et al., 2018). A bicistronic replicon has ubiquitin (host cleavable sequence) in-frame between reporter and selection marker helps in assessing inhibitory activity of antivirals (Figure 2E) (Vrolijk et al., 2003). Further, the replacement of nonstructural genes in replication-competent GT1b (Con1) and GT2a (JFH-1) replicons by corresponding non-structural genes of patient isolates from different GTs and subtypes resulted in the production of intergenotypic chimeras that have helped substantiate the identification of pangenotypic DAAs and resistance (Figure 2F) (Herlihy et al., 2008; Sheaffer et al., 2011; Liu et al., 2015; Ng et al., 2017b; Han et al., 2019). The molecular structures of bicistronic replicons shown in Figure 2D have been used as the basis to generate the intergenotypic replicons.

### Full-Length Bicistronic Replicons

Full-length bicistronic or genomic HCV replicons encode the entire HCV open reading frame core–NS5B and were generated for GT1b and GT1a that carried adaptive mutations (Figure 2G) (Pietschmann et al., 2002; Blight et al., 2003). The presence of *neo* also facilitated the selection of stable cell lines that support full-length RNA replication. However, the replication efficiency of full-length replicons was about 5-fold less than for subgenomic counterparts carrying the same adaptive mutations, and no evidence for infectious progeny release from hepatoma cells was observed. However, selectable JFH-1 full-length replicons are capable of producing infectious progeny (Date et al., 2007).

### Monocistronic Subgenomic Replicons

Monocistronic replicons lack EMCV IRES and consist of only HCV 5′ NTR that direct the translation of downstream viral coding sequences. Therefore, they closely resemble the structure of viral genomes (Figure 2H). Monocistronic replicons comprise the 5' NTR and first 12 codons of core sequence followed by the in-frame NS2-to-NS5B sequences. In this model, the cellular signaling peptidase mediates the cleavage between the capsid and NS2 (Blight et al., 2000). In other monocistronic replicons, the 5′ NTR translates the reporter gene that is in-frame with ubiquitin and the NS3-to-NS5B coding region, followed by the 3′ NTR (Figure 2I) (Reiss et al., 2013). (Frese, 2002) generated a monocistronic replicon containing the selection marker hygromycin phosphotransferase. In this case, cleavage between ubiquitin and NS3 is mediated by a cellular ubiquitin carboxyl-terminal hydrolase (Figure 2I). Monocistronic replicon replication can then be assessed by detection of viral proteins using non-structural protein-specific antibodies and viral mRNA with quantitative real-time RT-PCR.

### Replicon Systems in the Era of DAAs

The treatment strategies for HCV infections have radically changed in the last two decades, and particularly in the last 10 years (Cuypers et al., 2016). A better understanding of the molecular structure and function of hepatitis C proteins has especially allowed the design of antivirals that directly target non-structural proteins, including NS3/4A, NS5A, or NS5B, that facilitate RNA replication (Bartenschlager et al., 2013; Scheel and Rice, 2013). The NS3/4A inhibitor ciluprevir (BILN-2061) was the first developed DAA using the GT1a (Con1) replicon system and was tested in hepatitis C patients to demonstrate the proof-of-principle (Lamarre et al., 2003). However, ciluprevir was less effective against GT2 and GT3, emphasizing the dire need to develop efficient non-GT1 replicon systems. The non-GT1 replicon systems that have been developed have proven highly useful for testing the pangenoypic HCV replication inhibition activity by DAAs. Hence, HCV replicon systems have become indispensable in the preclinical research and development of effective DAAs, owing to advantages that include: (i) stable replicon cells easily integrate into high-throughput screening assays; (ii) it is reliable to assess and compare levels of HCV replicon replication; (iii) they are efficient for determining preclinical cytotoxicity; (iv) they are robust for determining pangenotypic antiviral activity and have a high genetic barrier to resistance; and (v) replicon systems yield no infectious progeny, thereby minimizing the risk of exposure unlike infectious HCVcc systems.

A candidate DAA would have desirable preclinical attributes that support its clinical development for HCV treatment. Since the identification of the first NS3/4A inhibitor, HCV replicon systems have been widely used to identify safe and effective pangenotypic antivirals. Among many effective, not limited to, the most effective DAAs that are recommended for HCV treatment include, NS3/4A protease inhibitors (PIs, names end in -previr): glecaprevir (ABT-493) (Ng et al., 2014), grazoprevir (MK-5172) (Summa et al., 2012), paritaprevir (ABT-450) (Pilot-Matias et al., 2015), and voxilaprevir (GS-9857) (Taylor et al., 2019); NS5A inhibitors (names end in -asvir): daclatasvir (BMS-790052) (Gao et al., 2010), ledipasvir (GS-9451) (Yang et al., 2014), elbasvir (MK-8742) (Coburn et al., 2013), velpatasvir (GS-5816) (Cheng et al., 2013), ombitasvir (ABT-267) (DeGoey et al., 2014), pibrentasvir (ABT-530) (Ng et al., 2014) and ruzasvir (MK-8408) (Tong et al., 2017); inhibitors of NS5B nucleoside polymerase (NPIs, names end in -buvir): sofosbuvir (GS-7977) and non-nucleoside (NNPIs): dasabuvir (?ABT-333) (Maring et al., 2009) (Figure 1). These DAAs exhibited subnanomolar 50% effective concentrations (EC_50_s) toward replicons expressing a wide range of HCV GTs with minimal cytotoxicity. Despite the limited inventory of druggable viral enzymes and corresponding low molecular weight DAAs exhibiting high efficacy, current DAA-based treatments have been highly effective for HCV elimination. However, the emergence of HCV resistance to DAAs in small patient populations (<5%) poses challenges to eradication (Popping et al., 2018). The crystal structures of NS3/4A, NS5A and NS5B are helping to solve the mechanisms of action of various DAAs and the molecular basis for DAA resistance (reviewed in Bartenschlager et al., 2013; Götte and Feld, 2016).

HCV treatment has dramatically improved since 2014 coinciding with the pegIFN and RBV-free co-administration of two or three pangenotypic DAAs that shorten treatment durations and lead to SVR rates above 95% in patients with chronic hepatitis C (EASL, 2018). It is worth to note that all currently recommended DAA regimen consists an NS5A inhibitor. The mode of action of DAAs at molecular level is not yet understood. HCV variants may harbor RASs that are associated with reduced susceptibility given the high mutation rate of HCV, and these may exist naturally and can be selected in patients due to the application of some DAAs. The biological and clinical implications of variant selection that are resistant to DAA and cause treatment failure are well-known (Lontok et al., 2015). Thus, insight into the nature of RASs and their mode of action is important to understand viral resistance to DAAs and to promote the eradication of HCV (Lontok et al., 2015; Cuypers et al., 2016; Wyles and Luetkemeyer, 2017). RASs are reported as “single letter amino acid code-number-single letter amino acid code.” For example, the NS5A-specific RAS Y93H manifest as the typically expected amino acid at position 93 of the NS5A protein in the predominant circulating virus has tyrosine (Y) and in some patients, viral variants may harbor the amino acid histidine (H) at position 93 of the NS5A protein. Sequencing technologies can be used to detect drug-specific RASs circulating in patients (Li and Chung, 2019). RASs present in at least 15% of all drug-target encoding viral sequences circulating in patients reduces the SVR for the patient (Pawlotsky, 2016). The number and type of RAS(s) required for a variant to remain fit in the presence of a DAA are referred to as the genetic barrier to resistance, which also differ according to DAA and class (Götte, 2012). A single RAS at a key position in the target protein can confer a low genetic barrier to resistance, while a high genetic barrier to resistance requires at least three RASs (McCown et al., 2008; Pawlotsky, 2011). The emergence of DAA resistance depends on the fitness of RASs and whether they exist at baseline in patients prior to treatment. The fitness of RASs determines the speed of resistant variant selection in the presence of DAAs (Feld, 2017). The individual NS5A-RASs M28T, Q30E/H/R, L31M/V, and Y93H/N are commonly found at baseline in 10–15% of GT1 treatment-naïve patients. These high fitness RASs to NS5A-inhibitors (e.g., daclatasvir, ledipasvir, and ombitasvir) confer a low barrier to resistance, and can emerge early and persist in patients following administration of NS5A-based treatments (Krishnan et al., 2015b; Zeuzem et al., 2017; Dietz et al., 2018; Fourati et al., 2018). The majority of these RAS are also selected in replicon cells and confer resistance to recently recommended pangenotypic NS5A-inhibitors (Cheng et al., 2013; Liu et al., 2015; Ng et al., 2017a; Asante-Appiah et al., 2018). In contrast, the NS5B RAS S282T exhibited reduced fitness and does not appear to emerge in patients following administration of sofosbuvir (NPI) (Lam et al., 2012; Wyles et al., 2017). Consequently, a typical goal of DAA-based combination regimens is to increase the genetic barrier such that resistant variants do not arise so readily (Chacko and Gaglio, 2015).

**Figure 3:**
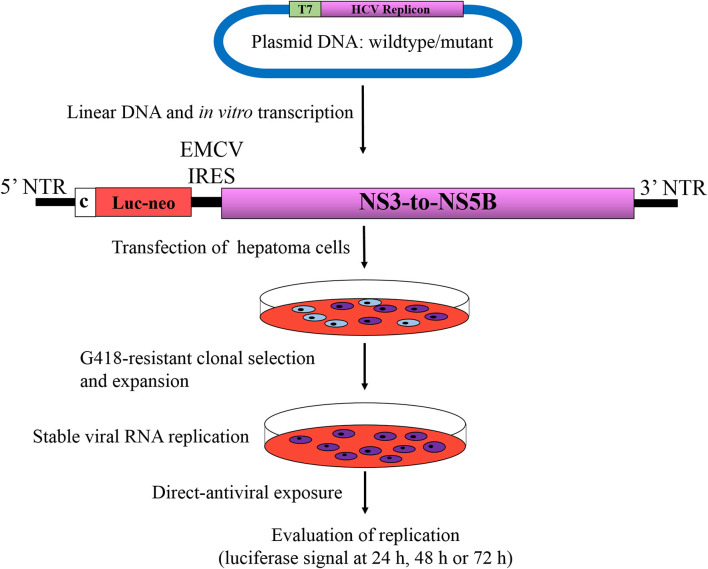
Phenotypic assay of HCV resistance to antivirals. Bicistronic replicons described in Figure 2D are the most widely used systems for determination of phenotypic direct-acting antiviral resistance. Replicon RNAs from wild type or mutant are synthesized by *in vitro* transcription of linearized plasmids harboring HCV replicon, which are then transfected into hepatoma cells, subsequently subjected to G418 selection pressure for 3–4 weeks. Cells harboring active replicon RNAs become resistance to G418 due to expression of *neo* gene, whereas untransfected cells or cells that do not support RNA replication will be eliminated during the process. After the establishment of stable cell line supporting HCV autonomous replication, cells are subjected to increasing antiviral concentrations based on of EC_50_ or EC_90_ values. The level of susceptibility is evaluated by measuring the luciferase activity at 24, 48, and 72 h after treatment, in comparison to its wild type counterpart, which is commonly expressed as fold resistance. Luc-Neo, fused luciferase and *neo* gene encoding neomycin phosphotransferase; c, core; EMCV IRES, internal ribosomal entry site from encephalomyocarditis virus. Viral and non-viral coding sequences are marked by rectangles; non-coding regions are indicated by horizontal lines.

Considering that any RASs that may confer resistance to a candidate DAA are not known or that they will likely rise, the replicon systems remain innovative, for instance, in the treatment of stable replicon cells with increasing doses of a DAA that may facilitate emergence of RASs (Bartenschlager, 2006). Another instance where replicon systems have proven useful is when new RASs might be selected for in patients following administration of a DAA. These new RASs could be introduced into the replicon by site-directed mutagenesis and are then challenged by DAAs at increasing doses based on EC_50_ or EC_90_ values for replication inhibition compared to the wild-type counterpart, which is commonly expressed as fold resistance (Figure 3). These two examples provide opportunities to examine the development of DAA resistance using replicon systems. Moreover, it is reasonable to assume that RASs conferring resistance to DAAs *in vitro* have clinical significance.

## Replicons of Genotypes 1–6 and Resistance-Associated Substitutions

### Genotype 1

HCV GT1 is the most globally widespread group and represents 46% of all infections. Subtype 1b is more prevalent than 1a, at 68 vs. 31%, respectively (Messina et al., 2015). Boceprevir and telaprevir (PIs) were the two first DAAs recommended for use in combination with pegIFN and RBV in patients infected with GT1 HCV in 2011. Although both PIs increased the SVR rate by about 30% in treatment-na?ve and -experienced patients compared to standard pegIFN and RBV, their low genetic barrier to resistance and highly adverse effects in GT1 patients (Pearlman, 2012; Wendt and Bourlière, 2013) led to the development of more effective PIs with higher genetic barriers to resistance. The pegIFN- and RBV-free DAA combination regimens targeting viral enzymes were developed in 2014 and have improved the SVR rate to above 90%, along with shorter treatment durations of about 8 or 12 weeks and minimal adverse effects (Afdhal et al., 2014; Kowdley et al., 2014). The currently recommended four highly effective DAA combination regimes, elbasvir/grazoprevir, glecaprevir/pibrentasvir, ledipasvir/sofosbuvir, or sofosbuvir/velpatasvir, exhibit SVR rates of 98%−100% (Afdhal et al., 2014; Kowdley et al., 2014; Feld et al., 2015; Forns et al., 2015). The amino acid substitutions V36M, T54S, Q80K/H/R, R155K/M, A156T/V, and D168E/V/Y/H/T confer resistance to different PIs in GT1 HCV patients. In addition, the NS5A-associated substitutions M28T/V, L28M/I, Q30H/R, L31M, and Y93H are the most prevalent in GT1 isolates (Zeuzem et al., 2017).

GT1a (H77) and GT1b (Con1) replicons, or their backbones in chimeric form, are extensively used to identify pangenotypic HCV inhibitors. Replicons harboring single NS3 RASs at positions 36, 43, 54, 80, 155, 156, or 168 or in combinations comprising two or three of the mutations have been tested for resistance to PIs. Both replicons with NS3 D168A/E/V conferred significant resistance to paritaprevir and grazoprevir, while A156T/V conferred resistance to glecaprevir. The replicon with NS3 R155K lost susceptibility to paritaprevir, but maintained susceptibility to glecaprevir and grazoprevir (Ng et al., 2017b). The RASs R155W, A156T, and D168K/L/R conferred 20- to 100-fold resistance, whereas A156/L/V exhibited >100-fold resistance to voxilaprevir (Han et al., 2019). The NS5A amino acid substitutions M28T/V and Q30D/E/H/R/K in the GT1a replicon yielded moderate to high resistance to ruzasvir, daclatasvir, ledipasvir, ombitasvir, elbasvir, and pibrentasvir, while L31M/V to GT1b, whereas Y93H/N/C in both the GT1 replicons conferred negligible to significantly high resistance (Krishnan et al., 2015a; Liu et al., 2015; Asante-Appiah et al., 2018). HCV GT1 replicons carrying amino acid substitutions that confer > 2-fold resistance to various DAAs are listed in the Table 1.

**Table 1:** Amino acid substitutions that confer resistance to NS3/4A, NS5A and NS5B inhibitors from HCV genotypes 1–6.

**Replicon genotype**	**Amino acid and position**	**Amino acid substitution(s) (> 2-fold resistance)**	**Direct-acting antiviral**
**NS3/4A**			
1a	V36	A	GRA/PTV/VOX/GLE/GZR
		C/G	PTV
		L	VOX/GZR
		M	GLE/PTV/GZR/VOX
		L	PTV/GZR
	F43	S	VOX
		A	GZR
	T55	I	VOX
		H	PTV/GZR
	Y56	K	GZR/PTV/VOX
	Q80	L	PTV
		R	GZR/GLE/PTV
		G	GZR/PTV
		K	PTV
	R155	I	PTV
		K	GRA/PTV/VOX/GLE/GZR
		M	PTV
		Q	GRA/PTV/VOX/GLE/GZR
		S/W	PTV
		T	GZR/PTV
		V	GZR
	R156	T	PTV
		S	GRA/PTV/VOX/GLE/GZR
		G	VOX
	A156	T	GLE
		L	VOX
	D168	C	GZR/PTV
		E	GLE/GZR/PTV
		G/T	GZR
		H	GZR/ PTV
		K	GZR/VOX
		L/R	VOX
		N	GZR/PTV
		V	GLE/GZR/PTV
		Y	GZR/PTV
		T	VOX
	I170	V	VOX
		A	GRA/PTV/VOX/GLE/GZR
1b	V36	C/G/K	PTV
		M/G	PTV/GZR
		S	GZR
		R	GZR/GLE/VOX
	Q80	K	PTV
	R155	K	GRA/PTV/VOX/GLE/GZR
		G	GZR/PTV/GLE
	A156	S/A	GZR/PTV
		T	GZR/PTV/GLE/VOX
		V	GZR/GLE/VOX
	D168	E/H/K/T/V/Y	GZR/PTV
		G/N	GZR
		Q	GRA
2	R123	V	GRA/PTV
	I132	A	GZR
	R155	K	GRA/PTV/VOX/GLE/GZR
		T	GZR/PTV
		G	GZR/PTV/GLE
		W	PTV/VOX
	A156	S	GZR/PTV
		V	GZR/GLE/VOX
		T	GLE/VOX
		A/E/G/H/N/V	GZR/PTV
	D168	V	GRA
	I170	F	GRA/PTV
	L175	R	VOX
3	K26	R/K	VOX
	Q41	A	VOX
	T54	R	VOX/GLE
	Q80	K	VOX
		A/C/F/T/Y	VOX
	S122	K	GRA/PTV/VOX/GLE/GZR
	R155	G	GZR/PTV/GLE
	A156	S	GZR/PTV/GLE
		T	GLE
	S166	A	GZR/PTV
	D168	V	PTV
		R	GRA/PTV
	Q168	R/H/I	VOX
		R	VOX/GLE
		L	GLE
	V170	T	GRA/ PTV
	I170	H/R	VOX
4	Q41	S	VOX
	T54	A/N/S/V	VOX
	T122	K	GRA/PTV/VOX/GLE/GZR
	R155	C	VOX/GLE/PTV/GZR
		G	GZR/PTV/GLE
	A156	S	GZR/PTV
		L/T/V	VOX/GLE
		S	VOX
	A166	E/K	VOX
	D168	V	VOX/GLE/PTV/GZR
		H	GLE/PTV/GZR
		I/L	VOX
	V170	V	GRA
5	E30	A/G/V	VOX
	T122	K	GRA/PTV/VOX/GLE/GZR
	R155	G	GZR/PTV/GLE
	A156	S	GZR/PTV
		S	VOX
	A166	A	GZR/PTV/VOX
	D168	E/H/V	GZR/PTV/VOX/GLE
		N	GZR/PTV
		K/R/Y	VOX
		V	VOX
		E	GLE
	I170	A	GRA/PTV/VOX/GLE/GZR
6	V36	K/R	VOX
	Q41	C	GRA/PTV
	F43	A	VOX
	V55	H	VOX
	Y56	K/Q/R	VOX
	L80	A/D/G/N/T	VOX
	S122	K	GRA/PTV/VOX/GLE/GZR
	R155	T	PTV
		G	GZR/PTV/GLE
	A156	S/I	GZR/PTV
	V158	A	GZR/PTV/VOX
	D168	E/V	GZR/PTV/VOX
		A/H	GLE/GZR/PTV
		G/N	GZR/PTV
		Y/V	VOX/GLE
**NS5A**			
1a	K24	G	LDV
		N	LDV
		R	LDV
	M28	A	DCV/LDV/ELB/OMV
		G	DCV/LDV
		T	DCV/LDV/OMV/PBT/ OMV/VEL/ELB
		V	OMV
	Q30	D	ELB
		E	DCV/LDV/OMV/PBT/ OMV/VEL/ELB
		G	LDV/ELB
		H	DCV/LDV/OMV/PBT/ OMV/VEL/ELB
		K	LDV/VEL
		L	LDV/VEL
		R	DCV/LDV/OMV/PBT/ OMV/VEL/ELB
		T	LDV
	L31	F	DCV/LDV/OMV/ELB
		I	LDV/VEL
		M	DCV/LDV/ELB/VEL
		V	DCV/LDV/OMV/PBT/ OMV/VEL/ELB
	P32	L	DCV/LDV/VEL
		S	DCV/LDV/VEL
	S38	F	LDV
	H58	D	DCV/LDV/OMV/PBT/ OMV/VEL/ELB
	A92	T	LDV
	Y93	C	DCV/LDV/OMV/PBT/ OMV/VEL/ELB
		F	LDV/VEL
		H	DCV/LDV/OMV/PBT/ OMV/VEL/ELB
		N	DCV/LDV/OMV/PBT/ OMV/VEL/ELB
		R	DCV/VEL
		S	LDV/VEL/OMV
		W	VEL
1b	L28	M	ELB
		T	DCV/OMV
		I	OMV
	R30	H	DCV
	L31	F	DCV/LDV/OMV/ELB
		M	DCVLDV/OMV/VEL
		V	DCV/LDV/OMV/PBT/ OMV/VEL/ELB
	P32	L	DCV/LDV/VEL
		S	DCV/LDV/VEL
	P58	D	LDV
		S	DCV
	A92	K	LDV
	Y93	H	DCV/LDV/OMV/PBT/ OMV/VEL/ELB
		N	DCV/LDV/OMV/PBT /OMV/VEL/ELB
		C	VEL/DCV
		S	VEL
2	F28	S	DCV/OMV
	L28	F	OMV
	L31	M	DCV/LDV/VEL
		V	DCV/LDV/OMV/PBT/ OMV/VEL/ELB
	C92	R	DCV
	Y93	H	DCV/LDV/OMV/PBT/ OMV/VEL/ELB
3	M28	T	DCV/LDV/OMV/PBT/ OMV/VEL/ELB
	A30	K	DCV/ELB/VEL
		T	DCV
	L31	F	DCV/LDV/ OMV/ELB
		M	DCV/LDV/OMV/VEL
		V	DCV/LDV/OMV/PBT/ OMV/VEL/ELB
	S62	L	DCV
	Y93	H	DCV/LDV/OMV/PBT/ OMV/VEL/ELB
4	L28	V	OMV
	L30	H	DCV
		R	VEL
		F	ELB
		S	VEL
	Y93	C	VEL
		H	DCV/LDV/OMV/PBT/ OMV/VEL/ELB
		N	DCV/LDV/OMV/PBT/ OMV/VEL/ELB
		R	DCV/VEL
5	L28	I	OMV
	L31	F	DCV/LDV/OMV
		V	DCV/LDV/OMV/PBT/ OMV/VEL/ELB
6	L31	M	DCV/LDV/ ELB/VEL
		V	DCV/LDV/OMV/PBT/ OMV/VEL/ELB
	P32	L	DCV/LDV/V EL
		S	DCV/LDV/VEL
	T58	A	DCV/OMV
		N	DCV/OMV
		S	DCV/OMV
**NS5B**			
1a	S282	T	SOF
	C316	H	DSV
		Y	DSV
		N	SOF
		F	SOF
	L320	F	SOF
	A395	G	DSV
	M414	T	DSV
	N444	K	DSV
	E446	K	DSV
		Q	DSV
	Y448	C	DSV
		H	DSV
	A553	T	DSV
	S556	G	DSV
		N	DSV
	D559	G	DSV
	Y561	H	DSV
	S565	F	DSV
1b	S282	T	SOF
	C316	H	DSV
		Y	DSV
		N	DSV
	L320	F	SOF
	S368	T	DSV
	N411	S	DSV
	M414	T	DSV
	Y448	C	DSV
		H	DSV
	P495	A	DSV
	A553	V	DSV
	G554	S	DSV
	S556	G	DSV
	D559	G	DSV
2	S282	T	SOF
	M289	L	SOF
3	S282	T	SOF

*GZR, grazoprevir; PTV, paritaprevir; GLE, glecaprevir; VOX, voxilaprevir; DCV, daclatasvir; EBR, elbasvir; LDV, ledipasvir; PBT, pibrentasvir; OMV, ombitasvir; DSV, dasabuvir; SOF, sofosbuvir*.

### Genotype 2

HCV GT2 infections comprised 8% of the patients with chronic hepatitis C virus in Europe (Mangia and Mottola, 2012). Treatment of HCV GT2 infections have historically resulted in higher SVRs than with HCV GT1 infections, even with lower doses of RBV and a shorter duration of therapy. SVR rates of 99% have been observed for HCV GT2 with sofosbuvir/velpatasvir or glecaprevir/pibrentasvir treatment in patients that previously failed therapy with pegIFN and RBV or a combined sofosbuvir and RBV treatment (Feld et al., 2015; Forns et al., 2017).

The efficient replication by the JFH-1 replicon makes it among the most extensively used replicon systems for identifying pangenotypic HCV inhibitors. The JFH-1 replicon carrying NS5A-F28S confers resistance to declatasvir, lidipasvir, and velpatasvir while L31V confers resistance to ombitasvir and Y93H to ruzasvir (Asante-Appiah et al., 2018). Several NS3 RASs confer significant resistance, including R155W to voxilaprevir and A156T/V to volxilaprevir and glecaprevir (Ng et al., 2017b; Han et al., 2019). The S282T mutation in the NS5B polymerase region is the only mutation that is associated with resistance to sofosbuvir and was identified in a 2b-infected patient that failed therapy during a clinical trial (Hedskog et al., 2015). HCV GT2 replicons harboring amino acid substitutions that confer > 2-fold resistance to various DAAs are listed in the Table 1.

### Genotype 3

The GT3 genotype accounts for 20–30% of all of the HCV infections globally and is the second most prevalent reported genotype in several East Asian and some European countries (Gower et al., 2014; Messina et al., 2015). Patients with GT3 infections have relatively faster rates of fibrosis progression in addition to higher incidence of steatosis and HCC when compared with individuals infected with other HCV GTs (Chan et al., 2017). Dual DAA regimens including sofosbuvir/velpatasvir and glecaprevir/pibrentasvir show effective SVR rates ranging from 94 to 100% and have thus become mainstays for treatment-naïve patients (Jacobson et al., 2017; Kwo et al., 2017; Zeuzem et al., 2018). The most commonly observed baseline NS5A RASs that confer high levels of resistance in GT3 infected patients are Y93H, A30K, and L31I/F (Lawitz et al., 2016; Wyles and Luetkemeyer, 2017). The RAS A30K is found in 87% of GT3b infected patients at baseline, while paired RAS A30K + L31M in 100% of patients infected with GT3b and GT3g (Bagaglio et al., 2019; Smith et al., 2019).

The GT3 bicistronic subgenomic replicon was constructed from the consensus sequence of the isolate S310 (SGR-S310) that yielded G418-resistant colonies. The inclusion of NS3 T235I, NS5A-T210A, and NS5B-R465K increased the replication potential of the parental replicon, rendering the replicon amenable for screening and evaluation of DAAs (Saeed et al., 2013). An intergenotypic GT3 replicon cloned in the Con1 genetic background that carried the RAS NS5A-Y93H mutation conferring resistance to different NS5A inhibitors, including daclatasvir, ombitasvir, and elbasvir, also conferred significant loss in susceptibility to the recently discovered pibrentasvir and ruzasvir (Ng et al., 2017a; Asante-Appiah et al., 2018). In addition, a stably replicating GT3a replicon (PR87A7) harboring NS3-168Q, which is a highly conserved amino acid among GT3a isolates, was found to impart resistance to several anti-NS3 DAAs (Guo et al., 2019). Recently, it is reported that RAS NS5b-A150V was shown associated with a reduced response to treatment with sofosbuvir and RBV, with or without pegIFN. Inclusion of A150V in NS5B of GT3a (S52) subgenomic replicon conferred resistance against sofosbuvir (Saeed et al., 2012; Wing et al., 2019). HCV GT3 replicons harboring amino acid substitutions that confer > 2-fold resistance to various DAAs are listed in the Table 1.

### Genotype 4

GT4 is responsible for more than 80% of all of the HCV infections in Africa and the Middle East, and GT4 infections account for nearly 20% of global infections (Nguyen and Keeffe, 2005; Kamal, 2011). Approved dual DAA regimens for GT4 treatment include glecaprevir/pibrentasvir, sofosbuvir/velpatasvir, elbasvir/grazoprevir, and ledipasvir/sofosbuvir (Curry et al., 2015; Feld et al., 2015). The recommended triple regimens comprise ombitasvir-paritaprevir-ritonavir and sofosbuvir-velpatasvir-voxilaprevir (Bourlière et al., 2017). Current HCV GT4 treatments exhibit SVR rates above 95% (Feld et al., 2015; Bourlière et al., 2017).

The GT4a replicon ED43 was constructed from the cDNA consensus sequence to facilitate the development of antivirals with pangenotypic activity (Gottwein et al., 2010). Although the wild-type bicistronic replicon (fused RLuc-Neo) failed to replicate, stable cell lines were established by the inclusion of the adaptive mutations NS5A Q34R and NS3 T343R in addition to NS5A-S232I, which was previously shown to enhance GT1b (Con1) replication (Peng et al., 2013). Incorporation of the mutation R465G (NS5B), which was also identified in Con1, combined with NS5A S232I yielded few G418 resistant colonies (Saeed et al., 2012). In the ED43 genetic background (carries adaptive mutations NS3 G162R) inclusion of the NS5A RASs L28V or L30H do not confer significant resistance against pibrentasvir, nor do Y93H or L30H against ruzasvir (Ng et al., 2017a; Asante-Appiah et al., 2018). However, incorporation of NS3-R156T/V and D168H/V in the stable chimeric GT4a replicon in a Con1 genetic background conferred resistance to glecaprevir, while NS5A L28V conferred resistance to ombitasvir (Krishnan et al., 2015a; Ng et al., 2017b). HCV GT4 replicons harboring amino acid substitutions that confer > 2-fold resistance to various DAAs are listed in the Table 1.

### Genotypes 5 and 6

Chronic hepatitis C due to GT5 or GT6 infections has low global prevalence. However, GT5 is widespread in Southern Africa, wherein up to 40% of individuals with chronic HCV have GT5 infections (Al Naamani et al., 2013). In contrast, GT6 is mainly concentrated in Southeast Asia, including in China, Vietnam, Thailand, and Myanmar, with a prevalence of ~30–40% (Luo et al., 2019). Due to limited numbers of patients with reported GT5 and GT6 infections, data for treatment efficacy are limited to small cohorts. FDA-recommended treatments for patients infected with HCV GT5 and GT6 include sofosbuvir combined with an NS5A inhibitor like velpatasvir or ledipasvir. Such treatments have achieved 95–100% SVR rates among treatment-naive patients with or without cirrhosis (Feld et al., 2015; Jacobson et al., 2017; Nguyen et al., 2019).

GT5a strain SA13 was isolated from the plasma of a chimpanzee infected with a South African patient sample (Bukh et al., 2010) and was used to develop an efficient GT5a replicon and intergenotypic replicons. Inclusion of the NS5A S232G adaptive mutation in the SA13 background has facilitated the generation of a stable replicon, but E176K, E196C, D405N/Y, and E533K have not (Camus et al., 2018). A replicon consisting of the NS5A coding sequence in the GT2a (JFH-1) background was recently synthesized to evaluate the antiviral activity of ruzasvir, which is a pangenotype NS5A inhibitor (Asante-Appiah et al., 2018). The RASs L18F, L31F, and L31S have frequently emerged as resistance-conferring mutations to ruzasvir (Asante-Appiah et al., 2018). Inclusion of the adaptive mutation NS5A S232I that was originally found in the GT1b Con1 replicon within the GT5a replicon [SA1/SG-neo(I)] moderately increases replicon replication compared to its wild-type counterpart. However, inclusion of NS3 E176K and K379S adaptive mutations in addition to the NS5A S393P mutation further enhanced replicon replication and facilitated screening of antivirals (Wose Kinge et al., 2014).

Gilead Sciences has generated GT6a subgenomic replicons containing the NS5A S232I adaptive mutation. Further, additional adaptive mutations including the NS3 mutations E30V and K272R and the NS4A mutation K34R enhances GT6a replicon replication. Inclusion of these new adaptive mutations allowed the establishment of an efficient replicon that was engineered with RLuc-Neo fusion to achieve reproducible quantification of HCV replication (Yu et al., 2014). Further, inclusion of the adaptive mutations NS3 K272R plus NS5A P237L enhanced the replication capacity of a GT6a replicon (GS16a-1) (Camus et al., 2018). In addition, a chimeric replicon consisting of the complete NS5A sequence GT6 in the GT2a (JFH-1) background was generated to evaluate the antiviral activity of ruzasvir (Asante-Appiah et al., 2018). The GT5a and GT6a replicons harboring the NS3 amino acid substitutions D168H/G/V and D168E/V/Y were associated with reduced susceptibility to glecaprevir and voxilaprevir, respectively (Ng et al., 2017b; Han et al., 2019). The RAS NS3-D168E was found in 48.6% of GT5 natural HCV sequences and 2.7% of those of GT6 (Patiño-Galindo et al., 2016). HCV GT5 and GT6 replicons carrying amino acid substitutions that confer > 2-fold resistance to various DAAs are listed in the Table 1.

## Conclusion

After the molecular cloning of HCV genome, establishing functional Con1 replicon constitutes one of the most significant steps in fundamental research on this pathogen of public health concern. Further improvements are accomplished by the advent of HCV replicons for most of the genotypes. HCV replicon systems are already proving valuable research tools for the discovery of effective pangenotypic DAAs, and to evaluate RAS(s) identified in both preclinical and clinical studies. It is not much of an exaggeration to say that HCV replicon systems have been and will continue to be the primary workhorses of the DAA discovery process and may provide a foundation for the elimination of HCV.

## Author Contributions

SK, SS, and NV contributed to the manuscript writing and final approval. All authors contributed to the article and approved the submitted version.

## Conflict of Interest

The authors declare that the research was conducted in the absence of any commercial or financial relationships that could be construed as a potential conflict of interest.
